# The availability of essential medicines for diabetes at health facilities in Bangladesh: evidence from 2014 and 2017 national surveys

**DOI:** 10.1186/s12913-022-07738-4

**Published:** 2022-03-22

**Authors:** Shariful Hakim, Muhammad Abdul Baker Chowdhury, Nasar U. Ahmed, Md Jamal Uddin

**Affiliations:** 1 Department of Statistics, Shahjalal University of Science & Technology, Sylhet, 3114 Bangladesh; 2 Chander Hat Degree College, Nilphamari, Bangladesh; 3 Department of Neurosurgery, College of Medicine, University of Florida, Gainesville, FL USA; 4 Department of Epidemiology, Florida International University, Miami, FL USA

**Keywords:** Availability, essential medicine, diabetes, Bangladesh

## Abstract

**Background:**

Bangladesh ranks among the world’s top ten countries in the number of diabetic patients. The prevention of this disease requires treating patients with essential medicines, and the first crucial step in the uptake of these medicines is availability. We aimed to assess the availability of essential medicines for diabetes (EM-Diabetes) and to explore health facility characteristics associated with the availability of those medicines.

**Methods:**

We performed the analysis using nationally representative data from the two waves of the cross-sectional Bangladesh Health Facility Survey (BHFS) in 2014 and 2017. Data are available for 1548 and 1524 health facilities in the 2014 and 2017 BHFS. Study samples of this study were 217 facilities (73 from 2014 and 144 from 2017) that offer diabetes diagnosis and treatment services. The outcome variable ‘EM-Diabetes availability’ was calculated as a counting score of the tracer medicines: metformin, glibenclamide, injectable insulin, and injectable glucose solution. A multivariable Poisson regression model was used to identify the health facility characteristics (such as, managing authority, location, external supervision, regular quality assurance activities, national guidelines for diagnosis and management of diabetes, etc.) associated with EM-Diabetes availability.

**Results:**

Since 2014, there have been minimal increases in Bangladeshi health facilities that provide diabetes screening and treatment services (from 4.7% to 9.4%). Among facilities offering diabetes services, 64.5% (BHFS 2014) and 55.7% (BHFS 2017) facilities had no EM-Diabetes on-site at all. Between 2014 and 2017, the availability of metformin increased (from 27.5% to 40.1%), but there was a decrease in the availability of glibenclamide (from 16.5% to 9.1%), injectable insulin (from 20.4% to 11.4%), and injectable glucose solution (from 20.4% to 19.2%). Furthermore, publicly owned facilities [relative risk (RR) = 0.44, 95% confidence interval (CI): 0.25–0.78 for 2014 and RR= 0.54, 95% CI: 0.41–0.71 for 2017] and facilities in rural settings [RR= 0.26, 95% CI: 0.12–0.55 for 2014 and RR= 0.60, 95% CI: 0.44–0.81 for 2017] were significantly associated with decreased availability of EM-Diabetes in both survey years. Moreover, routine user fees [RR=3.70, 95% CI: 1.86–7.38] and regular quality assurance activities [RR= 1.62, 95% CI: 1.12–2.34] were also significantly associated with increased EM-Diabetes availability in 2017 only.

**Conclusions:**

Overall, the health facilities in Bangladesh had insufficient essential medicines for treating diabetes. In general, the availability of EM-Diabetes declined from 2014 to 2017, except for metformin. Policymakers should consider a wide range of policy implications, focusing on the management of public facilities, rural facilities, routine user fees, and quality assurance activities to improve the availability of EM-Diabetes at health facilities in Bangladesh.

## Introduction

Diabetes mellitus is a leading cause of morbidity, mortality, and disability worldwide. An estimated 463 million people live with this condition, which will rise to 700 million by 2045 [1]. Diabetes was responsible for 4.2 million (11.3%) of global adult deaths in 2019 [1]. About 80% of the estimated total number of diabetic patients worldwide now live in low- and middle-income countries (LMICs), a trend expected to continue [1]. Moreover, diabetes causes a tremendous financial burden on the patient, the healthcare system, and the country’s productivity. These impacts will continue to grow substantially. Diabetes-related global health expenditure is estimated to reach $760 billion in 2019, rising to $825 billion by 2030 and $845 billion by 204 5 [2].

Diabetes has been implicated in several health complications, such as heart disease, stroke, renal failure, and blindness [3]. People with diabetes are at greater risk of adverse health outcomes than people without diabetes [4]. There is a prediction that more than half of diabetic patients will require at least one surgical procedure during their lifetime, and postoperative complications lengthen hospital stays, increase the economic burden, and increase mortality [5].

Among LMICs, Bangladesh ranks among the world’s top ten countries in terms of diabetic patients. The prevalence of diabetes among adults in Bangladesh is 9.2% (8 million); furthermore, an estimated 56% remain undiagnosed, and it is forecasted that the number of adults with diabetes will be 15 million by 2045 [1]. Like many other developing countries, Bangladesh is experiencing a disease burden switch from infectious diseases to chronic diseases [6, 7]. Of these, diabetes is one of the most prevalent diseases in Bangladesh.

The prevention of this disease requires tremendous governmental efforts, systems adjustment, creating a favorable environment, and individual behavioral change as a long-term plan. However, disease control and health maintenance remain the only viable options for millions of people with diabetes. This option requires treating patients with essential medicines, and the first crucial step in the uptake of these medicines is availability, followed by accessibility, acceptability, and quality [8]. In Bangladesh, both the public and private sectors provide health care. Most Bangladeshis generally get their medicine directly out-of-pocket (OOP) from the private sector [9].

Essential medicines to meet the majority of the population’s fundamental healthcare needs are required to always be available in adequate amounts to run health systems [10]. Assuring access to safe, efficient, quality, and affordable essential medicines and vaccines for all is one of the targets of the Sustainable Development Goals (SDGs) [11]. Despite being a vital element of the right to health, access to essential medicines to prevent and treat high-incidence diseases, including diabetes, remains low in LMICs [12–14].

The term “access” refers to a broad idea that includes five dimensions: availability, accessibility, accommodation, acceptability, and affordability [15]. Availability is an essential and challenging component, particularly in publically funded universal health care systems. It is the relationship between the type and quantity of items required and those offered. Continuous availability of essential medicines to provide high-quality health care services is a vital component of universal health coverage (UHC) [16]. Analysis of availability is often a proxy for assessing access to essential medicines in the context of public and universal health care systems [17]. For diabetic patients, long-term, frequently lifelong treatment demanding continuous medication is usual, which creates an availability of essential medicines (EMs) indispensable for treatment success and, in the end, survival [18].

In 2014, a study that investigated the availability of selected essential medicines for diabetes in LMICs revealed that the median number of essential diabetes medicines was 6, uniformly partitioned between insulin and oral medicines. Insulin analogs were selected as essential medicines by 20% of the countries. Among all the studied factors, an increase in the burden of diabetes and the wealth of countries were associated with the selection of higher numbers of essential diabetes medicines [19]. A current analysis of 30 surveys published by Ewen et al. found that the availability of diabetes medicines in the public sector was 16.7%, 20.8%, and 45.5%, respectively, in low-income, lower-middle-income, and upper-middle-income countries [20]. In the private sector, the percentages were 27.8, 23.1, and 31.8%, respectively. The medicines were not only available (in outlets with 80% availability) but also affordable (a maximum of 1 day’s wages of the lowest-paid inexperienced government employee to buy 30 days’ treatment). The Prospective Urban Rural Epidemiology (PURE) study demonstrated that the availability of metformin was 64.7%, 86.1%, and 88.2%, respectively, in pharmacies of low-income, lower-middle-income, and upper-middle-income countries. It was 10.3%, 29.3%, and 40.2% for insulin [13]. The study also found a significant association between availability and affordability with the use of diabetes medicines.

In 2019, a study by Kosonde et al. investigated the availability, price, and affordability of 61 medicines in Bangladeshi health facilities using the World Health Organization/Health Action International (WHO/HAI) survey methodology [21]. This study found that infectious disease medicines and non-essential medicines were more available than medicines for NCDs and essential medicines and mentioned the poor availability of medicines in the public sector. Moreover, some studies assessed the preparedness of health facilities for diabetes in Bangladesh [22, 23]. Biswas et al. found that among the facilities that offer diabetes services, only 0.4% had all four of the four service preparedness factors (guidelines, trained staff, equipment, and medicine) [22]. Another study indicated that diabetes service readiness was low, specifically in public and rural facilities in Bangladesh [23]. They also mentioned that facility type and basic amenity readiness level were the strongest predictors of readiness.

However, to our knowledge, no studies have sought to systematically study the availability of essential medicines for diabetes (EM-Diabetes) to explore and identify health facility characteristics associated with the availability of those medicines. To achieve the SDGs ensuring the consistent availability of EM-Diabetes at health facilities in Bangladesh, we need a better understanding of the factors influencing the availability of those medicines [24]. The present study sought to examine the availability of EM-Diabetes and its associated factors.

## Methods

### Study population and setting

This study used publicly available data from two waves of the Bangladesh Health Facilities Survey (BHFS), conducted in 2014 and 2017. This survey is also known as the Service Provision Assessment (SPA) survey, uses standardized questionnaires from the SPA component of the U.S. Agency for International Development (USAID)‘s Demographic and Health Surveys (DHS) Program. The survey was carried out by the National Institute of Population Research and Training (NIPORT) and the Ministry of Health and Family Welfare (MOHFW), with financial support from the Government of Bangladesh and USAID with technical assistance from ICF International, and the data was collected by the Associates for Community and Population Research (ACPR). The Demographic and Health Survey (DHS) administered the survey association with Bangladesh by the National Institute of Population Research and Training (NIPORT) with technical assistance from ICF, USA. The surveys used standardized questionnaires for facility inventory and health provider interview schedules. The details of the survey instruments are published elsewhere.

The samples for the 2014 and 2017 BHFS included all types of registered health facilities across the country’s all administrative divisions. The sampling frames were a list of 19,184 and 19,811 registered health facilities provided by NIPORT and MOHFW for the 2014 and 2017 BHFS, respectively. Stratified random sampling was applied to choose 1596 formal-sector health facilities for the 2014 BHFS and 1600 for the 2017 BHFS. For various reasons, including the fact that some facilities were closed or not functioning during the survey, interviewers did not survey some sampled facilities. Finally, data is available in the 2014 and 2017 BHFS for 1548 and 1524 health facilities. The details of the sampling procedure and study design have been illustrated elsewhere [9, 25].

### Selection of study sample

**Fig. 1:**
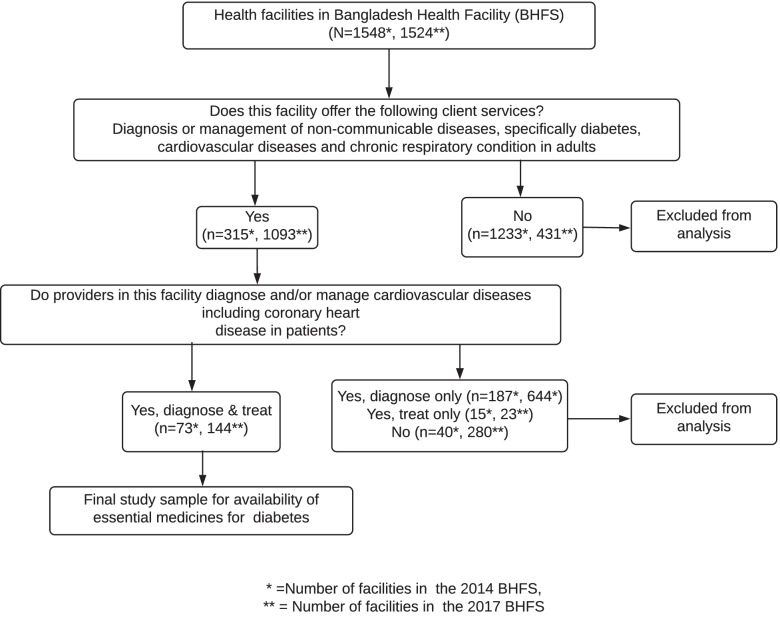
Flow Chart of Study Sample Selection

Study samples were 217 facilities (73 from 2014 BHFS and 144 from 2017 BHFS) that offer diabetes diagnosis and treatment services. Figure 1 depicts the screening process used to select study samples**.**

### Outcome variable

We evaluated the availability of EM-Diabetes using a list of tracer medicines (metformin, glibenclamide, injectable insulin, injectable glucose solution) according to the WHO-Service Availability and Readiness Assessment (SARA) reference manual [26]. Availability was marked out as the presence of at least one legal tracer medicine in a facility, visible during data collection, in keeping with direct observation of the data collectors. Essential drug availability measures the number of unexpired drugs in a health facility. The outcome variable ‘EM-Diabetes availability’ was calculated as a counting score of the tracer medicines ranging from 0 to 4, by which higher scores indicated the greater availability of essential drugs. The scores show how many of the four EM-Diabetes the facility had in stock on the survey day. Therefore, the outcome variable measures how many valid essential diabetes medicines are available in a health facility.

### Potential associated factors

Potential associated factors of interest include managing authority (public, private not-for-profit, private for-profit), location (urban, rural), administrative division (Barisal, Chittagong, Dhaka, Khulna, Rajshahi, Rangpur, Sylhet, and Mymensingh), external supervision (not received, received within the past 6 months, received more than 6 months ago), routine user fees or charges for client services (not available, available), 24-h staff coverage (not available, available), system to elicit clients’ opinions about the health facility or its services (not available, available), routine quality assurance activities (not performed, performed), inpatient care (not available, available), basic equipment score (low, high), diagnostic capacity score (low, high), and guidelines for the diagnosis and management of the disease (not available, available). The basic equipment involved in the domain score was as follows: adult scale, child or infant scale, thermometer, stethoscope, blood pressure apparatus, light source. Finally, the diagnostic capacity score included the following tracer items: urine pregnancy test, hemoglobin, blood glucose, urine dipstick (glucose), and urine dipstick (protein). The score for each facility is the sum of all the tracer items’ availabilities (i.e., value = 1) divided by the total number of items. The score variables are continuous, and we classified the distribution of scores into two equal parts. The facility had a low diagnostic capacity score if it scored up to a half (a score of 50%) and more than a half (> 50%) was considered a high. The cut-off point utilized in this study was also used to dichotomize the variable in prior investigations [27, 28].

### Data analysis

Using a chi-square test, we compared the proportion of EM-Diabetes availability between categories of various potential associated factors. A multivariable Poisson regression model was used to identify the health facility characteristics associated with EM-Diabetes availability. We used the variance inflation factor (VIF) to determine multicollinearity and omitted the variables—24-h staff coverage and inpatient care—from multivariable modeling since the VIF was greater than 3. Furthermore, we removed the variables-administrative division and external supervision from the modeling because of their low frequency (< 5%) in some categories. We utilized Stata 13 (StataCorp, College Station, TX, USA) for all data management and analysis. To account for the complex survey design, we weighted all our analyses using the weight option in Stata with the sampling weights provided in the dataset. In the modeling exercise, we utilized the “svy” command of Stata to account for the survey design, primary sampling unit, and cluster.

### Ethics approval

Our study was exempt from the ethical review approval because we used publicly available de-identified data from online data repositories. The Institutional Review Boards (IRB) of ICF International and Bangladesh Medical Research Council of Ministry of Health and Family Welfare (MOH&FW) has evaluated and approved the methodology and questionnaires for DHS surveys and assures that the survey complies with the U.S. Department of Health and Human Services regulations for the protection of human subjects (45 CFR 46).

## Results

### Univariate analysis

**Fig. 2:**
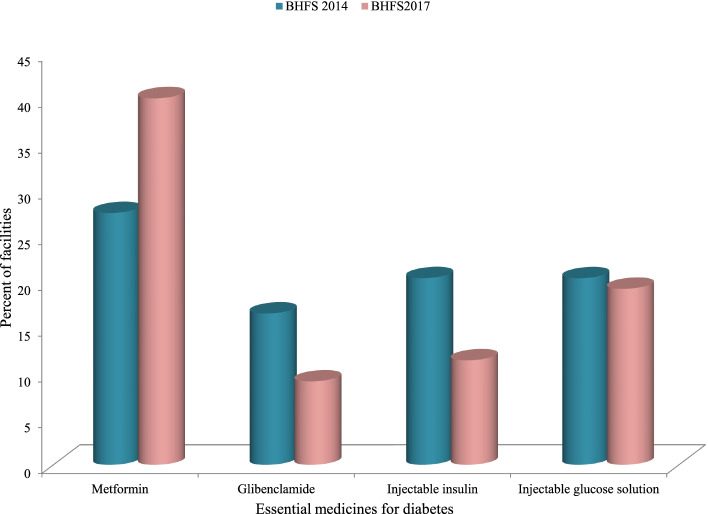
Availability of essential medicines for diabetes at health facilities in Bangladesh by the survey years

The count of various EM-Diabetes availability at every facility in the 2014 and 2017 BHFS was very skewed; scores were gathered at the lowest possible score and with a long tail towards the highest possible score (Fig. 2). Among facilities offering diabetes screening and treatment services, 64.4% (BHFS 2014) and 55.7% (BHFS 2017) facilities had no EM-Diabetes on-site at all.

**Fig. 3:**
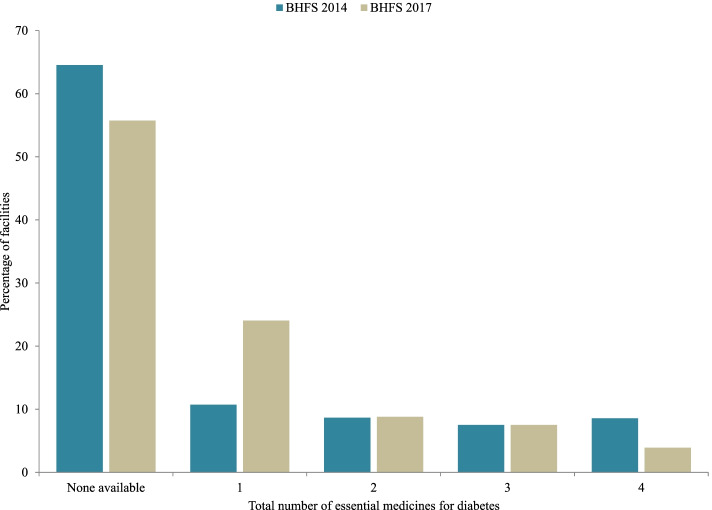
Distribution of number of essential medicines for diabetes in sampled facilities by the survey years

Figure 3 depicts the EM-Diabetes by category and the availability of those medicines at the sample of facilities in the 2014 and 2017 BHFSs, respectively. Between 2014 and 2017, the availability of metformin increased (from 27.5% to 40.140.1%), but there was a decrease in the availability of glibenclamide (from 16.5 to 9.1%), injectable insulin (from 20.4 to 11.4%), and injectable glucose solution (from 20.4 to 19.2%).

### Bivariate analysis

Table 1 shows the proportion of EM-Diabetes availability between the categories of various potential factors. In 2014, 85.9% of rural facilities and 59.9% of non-quality assurance facilities did not have EM-Diabetes, compared to 73.4 and 45.0% in 2017. Between 2014 and 2017, the availability of 1–2 EM-Diabetes has increased among public facilities (from 16.0 to 21.3%) and urban facilities (from 23.5 to 39.8%). There were only minimal decreases in the availability of 3 or more EM-Diabetes among the facilities that had routine user fees or charges for client services (from 24.0 to 15.6%), 24-h staff coverage (from 23.9 to 16.8%), and guidelines for diagnosis and management of diabetes (from 15.5 to 11.8%) since 2014.

**Table 1:** Distribution of different factors and their associations with EM-Diabetes availability

Facility characteristics	BHFS 2014 (***n*** = 73)				BHFS 2017 (***n*** = 144)			
	Number of available essential medicines for diabetes, n (%)				Number of available essential medicines for diabetes, n (%)			
	None available	1–2 available	3 or more available	***P***-value*	None available	1–2 available	3 or more available	***P***-value*
**Managing authority**				0.01				<0.001
Public	29 (82.4)	6 (16.0)	1 (1.6)		54 (77.1)	15 (21.3)	1 (1.6)	
Private	18 (48.0)	9 (22.6)	11 (29.4)		26 (35.3)	32 (43.9)	15 (20.8)	
**Location**				0.01				<0.001
Urban	22 (50.2)	10 (23.5)	12 (26.3)		30 (39.9)	30 (39.8)	15 (20.3)	
Rural	25 (85.9)	4 (13.3)	0 (0.8)		50 (73.4)	17 (25.1)	1 (1.5)	
**Administrative division**				0.77				0.52
Barishal	1 (62.1)	0 (17.3)	0 (20.6)		13 (83.6)	2 (13.1)	0 (3.3)	
Chattogram	13 (68.9)	3 (16.7)	3 (14.4)		14 (46.4)	12 (38.9)	5 (14.7)	
Dhaka	17 (60.1)	4 (15.0)	7 (24.9)		20 (49.5)	14 (36.5)	6 (14.1)	
Khulna	3 (57.4)	2 (27.8)	1 (14.8)		9 (67.5)	4 (29.4)	0 (3.1)	
Rajshahi	4 (66.0)	2 (28.8)	0 (5.2)		6 (43.9)	6 (44.2)	2 (11.9)	
Rangpur	5 (78.7)	1 (19.9)	0 (1.4)		7 (59.5)	3 (26.1)	2 (14.3)	
Sylhet	3 (63.3)	2 (34.8)	0 (1.9)		9 (67.0)	3 (23.4)	1 (9.6)	
Mymensingh					2 (36.4)	2 (48.7)	1 (14.9)	
**External supervision**				0.72				0.01
Not received	1 (39.5)	1 (18.4)	1 (42.1)		1 (28.7)	0 (4.1)	2 (67.2)	
Received within the past 6 months	38 (64.3)	12 (21.0)	9 (14.6)		69 (59.8)	37 (31.9)	10 (8.3)	
Received more than 6 months ago	8 (72.4)	1 (11.2)	2 (16.3)		10 (40.6)	10 (41.7)	4 (17.6)	
**Routine user fees or charges for client service**				0.01				<0.001
Not available	25 (85.7)	3 (10.1)	1 (4.2)		36 (92.6)	3 (7.4)	0 (0.0)	
Available	22 (50.4)	11 (25.6)	11 (24.0)		44 (42.3)	44 (42.1)	16 (15.6)	
**24-h staff coverage**				0.01				0.01
Not available	22 (94.4)	1 (5.6)	0 (0.0)		35 (76.4)	11 (23.6)	0 (0.0)	
Available	25 (50.1)	13 (26.0)	12 (23.9)		45 (46.0)	36 (37.2)	16 (16.8)	
**System to elicit clients’ opinions about the health facility or its service**				0.13				0.22
Not available	24 (76.1)	3 (10.1)	4 (13.8)		24 (66.8)	10 (28.4)	2 (4.8)	
Available	23 (55.5)	11 (26.6)	7 (17.9)		56 (52.1)	37 (34.3)	15 (13.6)	
**Routine quality assurance activities**				0.55				0.01
Not performed	24 (70.4)	6 (18.7)	4 (10.9)		41 (72.0)	14 (24.5)	2 (3.5)	
Performed	23 (59.9)	7 (19.2)	8 (20.9)		38 (45.0)	32 (37.9)	14 (17.1)	
**Inpatient care**				0.01				<0.001
Not available	25 (89.3)	3 (10.7)	0 (0.0)		48 (80.0)	12 (20.0)	0 (0.0)	
Available	22 (47.8)	12 (26.1)	12 (26.1)		32 (38.6)	35 (42.2)	16 (19.3)	
**Basic equipment score**				0.22				0.25
Low	8 (84.6)	2 (15.4)	0 (0.0)		6 (85.7)	1 (14.3)	0 (0.0)	
High	39 (61.6)	13 (20.0)	12 18.4)		74 (54.5)	46 (33.6)	16 (11.9)	
**Infection prevention score**				0.09				0.11
Low	19 (85.0)	2 (8.1)	2 (6.9)		18 (66.7)	9 (33.3)	0 (0.0)	
High	28 (55.2)	12 (24.5)	10 (20.3)		63 (53.7)	38 (32.4)	16 (13.9)	
**Diagnostic capacity score**				0.04				<0.001
Low	27 (78.8)	5 (14.2)	2 (7.0)		52 (73.2)	17 (24.0)	2 (2.8)	
High	20 (51.8)	9 (24.0)	9 (24.2)		28 (38.5)	30 (41.5)	14 (19.9)	
**National guidelines for diagnosis and management of diabetes**				0.71				0.49
Not available	30 (67.6)	7 (16.0)	7 (16.4)		68 (58.1)	36 (30.6)	13 (11.3)	
Available	18 (60.0)	7 (24.5)	5 (15.5)		12 (45.4)	11 (42.9)	3 (11.8)	

Due to the rounding of cell counts, percentages may not add up to 100%, and the number of valid cases may be different from the total count. Frequencies and percentages indicate weighted data

* *P*-value for χ2 test

### Multivariable analysis

Table 2 illustrates the result of the Poisson regression model for EM-Diabetes availability**.** In 2014**,** the likelihood of EM-Diabetes availability was 0.44 lower in public facilities than in private facilities (Relative Risk (RR): 0.44, Confidence Interval (CI): 0.25–0.78, *p*-value: <0.001). The 2017 survey yielded similar results (RR: 0.54, CI: 0.41–0.71, *p*-value: <0.001). The chance for EM-Diabetes availability was 74% lower in the facilities situated in rural areas compared to those in urban parts of the country in 2014 (RR: 0.26, CI: 0.12–0.55, *p*-value: <0.001). In the 2017 survey, similar finding was reached (RR: 0.60, CI: 0.44–0.81, *p*-value: <0.001). Moreover, facilities that charge routine user fees (RR: 3.70, CI: 1.86–7.38, *p*-value: <0.001) and conduct quality assurance activities (RR: 1.62, CI: 1.12–2.34, *p*-value: 0.01) had higher odds of EM-Diabetes availability in 2017.

**Table 2:** The relative risk (RR), 95% confidence interval (CI), and p-values for EM-Diabetes availability obtained from the Poisson regression model

Facility characteristics	BHFS 2014		BHFS 2017	
	RR (95% CI)	***P***-value	RR (95% CI)	***P***-value
**Managing authority**				
Private	Reference		Reference	
Public	0.44 (0.25, 0.78)	<0.001	0.54 (0.41, 0.71)	<0.001
**Location**				
Urban	Reference		Reference	
Rural	0.26 (0.12, 0.55)	<0.001	0.60 (0.44, 0.81)	<0.001
**Routine user fees or charges for client service**				
Not available	Reference		Reference	
Available	1.76 (0.82, 3.80)	0.15	3.70 (1.86, 7.38)	<0.001
**System to elicit clients’ opinions about the health facility or its service**				
Not available	Reference		Reference	
Available	1.10 (0.68, 1.77)	0.70	1.11 (0.78, 1.57)	0.57
**Routine quality assurance activities**				
Not performed	Reference		Reference	
Performed	0.88 (0.52, 1.47)	0.61	1.62 (1.12, 2.34)	0.01
**Basic equipment score**				
Low	Reference		Reference	
High	1.74 (0.69, 4.42)	0.24	0.86 (0.43, 1.75)	0.68
**Diagnostic capacity score**				
Low	Reference		Reference	
High	1.20 (0.64, 2.24)	0.56	1.37 (0.98, 1.91)	0.06
**National guidelines for diagnosis and management of diabetes**				
Not available	Reference		Reference	
Available	1.17 (0.72,1.92)	0.52	0.92 (0.60, 1.42)	0.71

## Discussion

The goal of the present study was to assess the availability of EM-Diabetes and explore what factors influence the availability of these medicines at different health facilities in Bangladesh. In general, the availability of EM-Diabetes declined between 2014 and 2017, and all of the medicines were insufficiently available at health facilities in both survey years. We found a significant association between the managing authority of the facility, location, routine user fees or charges for client service, and regular quality assurance activities with EM-Diabetes availability.

Essential medicines should be available at a health facility when patients come in with their prescriptions. But, we found that many facilities, particularly those under public management, had no EM-Diabetes on-site at all during the survey. It could be due to a lack of supply, poor stock management, a shortage of experienced pharmacists, medicine theft, incompetent transport, and distribution systems, or deferred stock level ordering and monitoring [29–32]. The government should make the required efforts to ensure that Bangladeshi health facilities have computerized medicine supply management systems, timely procurement, continuous supply chain management, and trained pharmacists. Moreover, we observed a change in the availability of EM-Diabetes from 2014 to 2017 at health facilities in Bangladesh. Compared with 2014, the availability of injectable insulin and glibenclamide decreased in 2017, while the availability of metformin increased. Producers’ insufficient inducements for making EM-Diabetes, whose price was set very low, could be one cause of the downward trend [33]. On the other hand, metformin is less expensive than insulin and may be easier to take.

Our findings imply that private facilities are more likely to have EM-Diabetes when compared to public facilities. It is particularly essential since medicines in public facilities are funded and dispensed for free but are only available in private facilities for a charge [34], and patients obtain them from private facilities, putting them at an increased financial burden. The lack of essential drugs in the public sector could be due to the planned medicine hierarchization for public sector procurement; nevertheless, the private sector appears to have also been influenced. Private facilities are not funded and rest on earnings from clients, so they will be more willing than public facilities to offer quality services and better fulfill patients’ healthcare requirements [35]. Another cause could be that certain government staff, despite having a large stock of medicines that are supposed to be free of charge at government hospitals, unlawfully sell them to private hospitals. Therefore, the poor availability of essential medicines, particularly in the public sector, requires more concentration and endeavor by the relevant authorities to confirm that these medicines are stocked in facilities.

Prior research has revealed significant disparities in the availability and provision of health care services between urban and rural facilities [27]. In keeping with the previous studies, rural facilities were less likely to have high EM-Diabetes availability than urban facilities. This finding could be attributable to the fact that rural residents’ healthcare needs differ from those in urban locations, and they face healthcare challenges that limit their capacity to receive the care they require [36]. Moreover, limited funding and remaining resource constraints affect several health facilities in remote and rural regions, causing incompetence in managing supply logistics systems to lead to the poor availability of essential medicines and equipment [37]. Therefore, rural facilities’ access to necessary and suitable healthcare services, which must be supplied and accessible on time, should be a priority for policymakers and health service planners.

In both 2014 and 2017, we observed that facility administrative division was a significant predictor for EM-Diabetes availability. The availability of EM-Diabetes varies among the different divisions in Bangladesh. When compared to facilities in Dhaka in 2014, facilities from all other divisions had a lower percentage of EM-Diabetes availability. In 2017, all other divisions had a higher percentage of EM-Diabetes availability than facilities in Dhaka, with the exception of Barishal and Khulna. This may be because the provision of diabetes services has exhibited notable increases in all divisions [25], and hence these facilities are more likely to be furnished with EM-Diabetes. Moreover, since the reasons for these differences are still unknown, further studies may be conducted to examine the underlying causes of this variation among the health facilities in Bangladesh. Moreover, targeted investments are needed to fortify service provision in divisions with low availability of EM-Diabetes.

Quality assurance (QA) is a procedure that focuses on assuring and maintaining a high level of the service offered in various health care facilities [32]. The findings of this study indicate that routine QA activities were significantly associated with EM-Diabetes availability in 2017, which is consistent with findings from studies conducted in Tanzania and Bangladesh [29, 31]. This study also showed that facilities that performed routine QA activities had a higher possibility of having EM-Diabetes than those that did not. This could be because QA entails continual monitoring and feedback to enhance the services provided [30]. Consequently, these facilities are more likely to enhance service availability based on QA team suggestions, which could lead to higher medicine availability. Nonetheless, the uptake of QA activities in Bangladeshi health care facilities remains low because only a few facilities have routine QA activities and documentation of such activities [9, 25].

Partial or full payment for health services by health consumers is a policy option that several developing nations have used to raise funds to meet the growing demand for health care services [30]. According to the findings of this study, the availability of EM-Diabetes was significantly associated with the facility’s routine user fees in 2017, where facilities that charge regular user fees or costs for client services have a higher chance of EM-Diabetes availability than those that do not. User fees are a way for health systems to improve the quality of health services and expand the services available with the additional funds, potentially resulting in the high availability of medicines. Affordability data was not available for this study. In Bangladesh, EM-Diabetes is delivered free of charge in public facilities, but only private facilities are assessed for affordability. According to a recent study, a patient with diabetes, hypertension, and hypercholesterolemia might spend approximately five days’ salary on medications [21]. As a result, Bangladesh has a high rate of catastrophic healthcare costs. In Bangladesh, diabetes raises morbidity and mortality rates, reduces the quality of life, and adds to increased healthcare expenditures and burden [38, 39]. To improve diabetes management and avoid complications, enhanced access to healthcare services is needed [40]. Lack of healthcare professionals (who can diagnose and efficiently treat diabetes) and limited healthcare capability impede diabetes care. Moreover, a shortage of a continuous supply of medicines and equipment is also among the obstacles to efficient diabetes care. Carrying out a multi-sectoral strategy and finding cost-effective prevention approaches are essential to enhancing diabetes care in Bangladesh [41].

The Service Provision Assessment (SPA) survey/health facility survey is a health facility evaluation that provides a thorough overview of a country’s health service delivery. SPA surveys fill a critical gap in developing countries’ efforts to strengthen their healthcare systems. They collect data on the overall availability of various facility-based health services and their readiness to provide those services. In 2012, the SPA questionnaires were updated in collaboration with global agencies to make them easier to use and incorporate more information. In the new questionnaire, almost all the variables in one country’s SPA are identical to those in other nations’ SPAs. As a result, a range of developing countries can apply the findings of this study, including Afghanistan, the Democratic Republic of the Congo, Haiti, Malawi, Nepal, Senegal, Tanzania, and others. Understanding the availability of essential medicines and health facility characteristics associated with the availability of these medicines is critical for health systems and individuals in Bangladesh and other developing countries to meet the unprecedented burden of diabetes in the coming decades. Our findings could help guide strategies for improving access to EM-Diabetes in high-need sectors in Bangladesh and other developing countries, as the continuous availability of essential medicines is a critical component of universal health coverage (UHC).

### Strength and limitations

This study has several strengths. First, to our knowledge, this study is the first of this nature in Bangladesh, giving an insight into the essential medicine available for diabetes. This study used a nationally representative sample of health facilities in Bangladesh in which our findings reveal important information about the factors responsible for medicine availability for treating diabetes. Second, we adjusted the estimates for cluster effects and sample weights because SPA data was collected using a complex sampling strategy. Finally, we conducted a comparative study using the 2014 and 2017 BHFS, whereby the changes in EM-Diabetes availability among health facilities in Bangladesh can be observed.

There are some notable limitations to our approach. First, data was collected at a specific point in time, so there is no variation in availability over time. The current study is incapable of inferring causality. Longitudinal research is required to understand the factors better associated with the availability of EM-Diabetes. Second, we were unable to address access in a direct way, which is dependent on both availabilities and costs because BHFS does not collect data on EM costs. Third, BHFS collected data on how many medicines a specific facility had in stock on a single day. This procedure fails to explain stock disparities over time. At last, since there are many zeros for outcome variables, we ignore zero-inflated count models in this study.

## Conclusion

Overall, only a few health facilities in Bangladesh had adequate essential medicines for treating diabetes. The availability of EM-Diabetes declined between 2014 and 2017, and all of medicine was insufficiently available in both years. Patients with diabetes need an adequate and consistent supply of essential medicines. There is a range of policy options that government authorities should consider: the emphasis on public facilities, rural facilities, routine user fees, and quality assurance activities to bolster the healthcare system in Bangladesh for better availability of EM-Diabetes and restrain their rising effect on health outcomes.

## Data Availability

All data is publicly available in dhsprogram website (www.dhsprogram.com).

## Abbreviations

BHFS: Bangladesh Health Facility Survey
NCD: Non-communicable disease
WHO: World Health Organization
SDG: Sustainable development goal
LMIC: Low- and middle-income countries
NIPORT: National Institute of Population Research and Training
RR: Relative Risk
